# Workshop on: Chemistry of Metals in Medicine - The Industrial Perspective

**DOI:** 10.1155/MBD.1997.119

**Published:** 1997

**Authors:** Peter J. Sadler

**Affiliations:** Department of Chemistry University of Edinburgh West Mains Road Edinburgh EH9 3JJ UK

## Abstract

The Workshop was attended by 61 participants from 20 countries. Most of the speakers were industrialists and the Chairpersons and Discussion Leaders were academics.

The area “Chemistry of Metals in Medicine” has the potential for producing innovative, high quality, and original research.

This is a new and emerging area of biomedical chemistry. Small firms are already being established which are devoted to the new elemental medicine. Major pharmaceutical and healthcare industries too are becoming aware of the major impact which metal chemistry is likely to have on traditional organic pharmacology and of the new opportunities which it presents for advances including the development of metalloenzyme-specific inhibitors, targeted radionuclide complexes for diagnosis and therapy, contrast agents for magnetic resonance imaging, safer mineral and vitamin supplements, new agents for the treatment of neurological, gastrointestinal and cardiovascular disease, skin conditions, cancer, and microbial and viral infections.

The European scientific and technological research base in this area is potentially attractive for business. Industrial collaboration and cooperation can be accommodated within the COST framework.


### COST ACTION D8 (1996-2001)

Workshop

### Chemistry of Metals in Medicine - The Industrial Perspective

### 22-24 January 1997
### Louvain-la-Neuve, Belgium

### Report

**Summary**
The Workshop was attended by 61 participants from 20 countries. Most of the speakers were industrialists and the Chairpersons and Discussion Leaders were academics.

The area "Chemistry of Metals in Medicine" has the potential for producing innovative, high quality, and original research.

This is a new and emerging area of biomedical chemistry. Small firms are already being established which are devoted to the new elemental medicine. Major pharmaceutical and healthcare industries too are becoming aware of the major impact which metal chemistry is likely to have on traditional organic pharmacology and of the new opportunities which it presents for advances including the development of metalloenzyme-specific inhibitors, targeted radionuclide complexes for diagnosis and therapy, contrast agents for magnetic resonance imaging, safer mineral and vitamin supplements, new agents for the treatment of neurological, gastrointestinal and cardiovascular disease, skin conditions, cancer, and microbial and viral infections.

The European scientific and technological research base in this area is potentially attractive for business. Industrial collaboration and cooperation can be accommodated within the COST framework.

---

*The* ***COST*** *programme provides funds to allow concertation of research work in 25 European countries (and others if there is justified mutual benefit). The research work itself is funded by national bodies.*
***COST*** *is valuable because it promotes: working as a European team, development of synergy in research activities, establishment of an effective critical mass, avoidance of duplication of work, rapid dissemination of results, the establishment of a strategy for fundamental research in Europe, coordination of scientific policies, optimisation of European mobility.*

---

Papers presented

***An academic view of metals in medicine***
***P.J. Sadler (Edinburgh, UK: Chairman COST D8)***

Ninety elements occur naturally on earth. Of these, 9 are radioactive, and the remaining 81 could support life; 61 are metals. It is believed that Na, Mg, K, Ca, V, Mn, Fe, Co, Ni, Cu, Zn, Mo and Sn are essential for mammalian life. On the one hand, major advances in the understanding of the biological chemistry of some of these metals have occurred over the past few years (e.g. the structures of Fe, Cu and Zn metalloproteins and metalloproteins) which can be exploited in medicine and health care, whereas on the other hand our understanding of the physiological roles of some metals is very poor (e.g. Mn, Sn), and indeed there may be additional metals that are essential to life. Both essential and non-essential, natural and man-made elements can be used effectively in therapeutic and diagnostic medicine.

This COST Action includes 5 main themes.

*Metallodrugs in clinical use :* the exploration of the structures and chemical mechanisms of action of anticancer, antiarthritic, antimicrobial, antiulcer agents and antacids (e.g. containing Li, Mg, Al, Zn, Pt, Au, Bi), as well as mineral supplements. This can include problems of formulation, storage, the optimisation of administration, combination therapy, reduction in side-effects and use of rescue agents, as well as molecular mechanism of action.

*Radiopharmaceuticals and imaging agents :* including the development of radionuclide complexes for diagnosis and therapy, and contrast agents for magnetic resonance imaging (MRI). This theme involves the design of ligands to control the thermodynamic and kinetic properties of metal complexes, and to achieve functional diagnosis.

*Metalloproteins, enzymes and prosthetic groups :* this theme promotes the design of specific inhibitors of metalloenzymes, the understanding of DNA coding for metalloproteins and homeostasis, role of metals in controlling transcription, and use of coenzymes in diagnosis and therapy. Small metal complexes which mimic metalloenzymes could also find use as drugs.

*Speciation of metal compounds :* this is an important section because speciation controls the biological activity of metal ions (activity and toxicity). Studies will include those on oxidation states, types and numbers of coordinated ligands, heterogeneous media (including biofluids, cells, tissues, and nanosurfaces), establishment of data bases. Metal-ion specific and cell-specific chelating agents offer scope not only for the treatment of metal-related diseases but also as combination agents for organic drugs.

*Design and synthesis:* novel compounds will be designed, for example with low toxicity, incorporating features such as ligand and metal-based chirality, unusual oxidation states, and uncommon metals. New delivery systems, slow release agents, the establishment of structure-activity relationships, as well as molecular modelling and computation, are all included in this section of the programme.

***The industrial future for metals in medicine***
***M. Abrams (AnorMED)***

AnorMED is a new company (a spin-off from Johnson Matthey) based in Canada and devoted entirely to “Elemental Medicine”. Their potential products include AMD3100, a metal chelating bicyclam with potent antiHIV properties which inhibits the entry of the virus into cells by binding to virus coat protein gp120, and AMD473 a sterically-hindered 3rd generation platinum complex active against refractory disease and resistant tumours.

It must be emphasised that *for inorganic compounds, just as for organic compounds, activity, toxicity and pharmacokinetics are related to structure.* By appropriate design of the ligands, fine control over metal reactivity can be obtained. The natural properties of metal ions can be utilised in pharmacology: thus ruthenium complexes can control the nitric oxide levels and have potential for blood pressure control

A wide range of diseases may be treatable via specific inhibition of important metalloenzymes. These include: angiotensin-converting enzyme, matrix metalloproteinases, ribonucleotide reductase, methionine synthetase, nitric oxide synthase, carbonic anhydrase and cytochrome P450.

***Metal ions in neuroscience***
***I. Ragan (Merck, Sharp and Dohme)***

*Metal ions play an important role in neuroscience, being implicated in acute and chronic neurodegenerative disorders.* $Zn^{2+}$ and $Ca^{2+}$, for example, are implicated in brain damage following stroke. These metal-induced processes may be preventable by chelation therapy.

In Parkinson’s disease, iron may be responsible for oxidative stress, production of hydroxyl radicals, and loss of dopaminergic neurons. The role, if any, of $Al^{3+}$ in Alzheimer’s disease is unclear.

In some European countries the proportion of the population taking lithium as prophylaxis for bipolar depression is as high as 0.1%. Plasma Li concentrations of 0.5 to 0.8 mM are reached (ineffective at lower doses, toxic at higher doses). The mechanism of action of lithium is not clear, but may involve inhibition of the phosphoinositide cycle. The abnormalities caused by lithium in developing frog embryos appear to result from inhibition of glycogen synthase kinase, whose important role in cell signalling may point to its involvement in the therapeutic action of lithium.
Organomanganese complexes such as Eukarion's EUK-8, catalyze superoxide dismutation and breakdown of $H_2O_2$. Such compounds are in development for disorders resulting from oxidative stress mediated by reactive oxygen species.

***The impact of delivery systems on the use of metals in medicine***
***J.S. Fox (Shire Pharmaceuticals)***
Shire Pharmaceuticals was established in 1986 and specialises in drug delivery technology. *There is scope for improving current delivery systems for metal compounds of known therapeutic benefit, and also to identify novel therapeutic uses of metals, and link potential uses to enabling technologies.* New technologies include the use of viral vectors, liposome encapsulation, monoclonal antibodies, biodegradable polymer implants. Rare earth metal complexes are being developed as phosphate binding agents.
There are opportunities to achieve reduction and control of metal toxicity via targeted delivery and targeted action of metals, leading to higher efficacy and safety profiles.

***Iron therapy and oxidative stress***
***P.Geisser (VIFOR)***
VIFOR is a world-leading Swiss-based company in the field of oral and parenteral iron preparations. Despite the essentiality of iron, iron compounds must be used carefully in therapy. They can have serious toxic side-effects. Iron salts should not be used in critically ill patients, nor in combination with vitamin C. *It is important to design and use $Fe^{3+}$ compounds with low redox potentials to minimise risk of $Fe^{2+}$ medicated free radical reactions, especially in the case of pregnancy, prematurity, and iron deficiency anaemia, where a high underlying oxidative stress is observed.* Examples of such $Fe^{3+}$ compounds include iron-dextran, iron-dextrin (iron polymaltose), iron-sucrose - polynuclear $Fe^{3+}$ complexes related to ferritin. Careful studies are required of iron compounds in food. The speciation of metals in food is a field closely related to medicine.

***Development of novel platinum-based anticancer drugs***
***K.R. Harrap (Institute of Cancer Research)***
The Institute of Cancer Research was closely involved in the development of the second generation anticancer drug carboplatin in collaboration with Bristol Myers and Johnson Matthey. This successful partnership has also since developed the orally active Pt complex JM216.
The Institute is now involved with a new sterically-hindered Pt complex AMD473 in collaboration with AnorMED.
The aims of current work are to achieve: activity against refractory and relapsed disease (acquired resistance, especially ovarian cancer), efficient absorption form the gastrointestinal tract (oral availability), efficient uptake in cisplatin transport-impaired cells, reduced inactivation by thiols, and an altered DNA binding profile compared to cisplatin.
Certain platinum complexes are neurotoxic and this needs to be understood. It is closely related to the structures of the complexes. For example some complexes containing DACH (1,2-diaminocyclohexane) as a ligand are neurotoxic.
These successful Pt-based drug discovery programmes have shown that *drug discovery should be chemistry driven.* There is a need for the design of metal compounds with increased selectivity and specificity.

***Gold compounds***
***G. Leonard (Smith Kline Beecham)***
Over 500 gold compounds have been used medically. Five are in current parenteral use as a second-line therapy for difficult cases of rheumatoid arthritis: aurothioglucose, aurothiomalate, aurothiopropanol sulphonate, aurothiosulphate and aurothioglycanide. The lipophilic complex auranofin is the only oral gold drug on the market. Release is the key to the formulation - auranofin is marketed in a specially designed tablet containing lactose, starch, cellulose, starch glycollate and Mg stearate. *The mechanism of action of gold is poorly understood and requires further study.*

***Radiometals in diagnosis and therapy***
***J. Burke (Amersham International)***
Radionuclides can be used for diagnosis (gamma ray imaging) and therapy. There is a general move towards Tc-based imaging agents. Use of radionuclides in therapy is much less advanced than in imaging.
New Tc agents have been made which are responsive to in vivo reduction potentials (imaging of hypoxic regions), and agents can be designed which cross the blood brain barrier and are trapped in the brain.
A future aim is to target imaging agents to specific cell receptors (e.g. neuroreceptors). Targeting of metal complexes to nuclei of tumour cells could result in selective therapy.
*Determinations of the structures of the administered complexes and a plausible mechanism of action are necessary today for credible licence applications in this field.*
$^{89}SrCl_2$ (Metastron) provides a good example of effective targeted radioisotope (treatment of bone pain in metastatic bone disease). Clinicians require a range of metal ions with different half-lives and radiochemical properties. Some potential applications are limited by the availability of the isotopes from existing nuclear reactors: e.g. Re, Ru.
Smart synthetic methods often required for complexes of short-lived isotopes.

***X-Ray and magnetic resonance imaging (MRI) contrast agents - outlook in diagnostic imaging***
***F. Uggeri (Bracco)***
Ideally contrast agents should be completely non-toxic and all the administered dose is recovered.
*Four gadolinium MRI contrast agents are now on the market.*
It is possible to design hepatospecific MRI contrast agents based on Mn (e.g. Mn-DPDP) or Gd (e.g. Gd-BOPTA). The use of iodinated ligands to confer hepatospecificity on Gd complexes is being explored. Dendrimer complexes offer possibilities for attachment of several Gd centres.

***Properties of paramagnetic metals in (MRI)***
***M. Shaeffer (Guerbet)***
There is much industrial activity in this field. About 4 large companies and 10 small companies currently in the field of MRI contrast agents and 7 agents have been approved for clinical use.
Superparamagnetic agents have been designed consisting of colloidal iron oxide particles surrounded by a polymer coat. Some are blood pool agents and others taken up by the liver. The sizes of the particles determine the pharmacokinetics and their paramagnetism.
Targeting likely to be the key to future designs. *Basic research into increasing the paramagnetic efficiency of metal compounds would be of great help to industry.*

***Antiviral metal complexes***
***E. De Clercq (Rega Institute)***
Organic agents alone have not provided a cure for HIV. *There is a need for fundamental research on antiviral metal compounds and metal chelating agents combined with modeling, mechanism of action studies and the establishment of structure-activity relationships*
Metal compounds can inhibit virus entry into cells by binding to e.g. gp120 protein, polyanions such as Keggin- and Dawson-type polyoxometallates are active at low doses and have a low toxicity.
Anionic metalloporphyrins are also active against HIV and other viruses (less toxic with metal present).
The bicyclam JM3100, a metal chelating agent, is one of the most potent inhibitors of the HIV virus ever described, and appears to target a loop region in gp120. The presence of $Zn^{2+}$ markedly increases the binding of the bicyclam to the virus.
A Texas company (Aronex) is developing an oligonucleotide which forms a tetraplex structure (two G quartets and $K^+$ for stabilization), and interferes with viral DNA integration probably through binding to a protein.
Resistance is a problem for antiviral agents (virus changes coat protein sequence etc) and combination therapy may have potential.

***Inhibition of matrix metalloproteinases as an approach to disease therapy***
***P. Beckett (British Biotech)***
These enzymes are destructive enzymes implicated in e.g. cancer, arthritis, multiple sclerosis, cardiovascular disease, inflammatory bowel disease and osteoporosis. *Consequently over 30 companies are involved in this area of pharmaceutical research.*
After about 10 years of work, British Biotech has introduced the oral peptidomimetic Marimastat into clinical trial. This drug contains an N-terminal hydroxamate group which effectively mimics the protein amide bond to be cleaved. Marimastat is now in advanced stages of clinical trials (phase III) for cancer treatment.
*Targeted enzyme-specific metal chelating agents may find application for inhibiting other important metalloenzymes and provide the basis for novel drugs.*

***Development of orally-active iron chelators for clinical use***
***H.P. Schnebli (Novartis Pharma)***
The currently used agent for treatment of iron overload, Desferral, is inactive orally and has a short duration of action ($T_{1/2}$ of 12 min in plasma, continuous infusion needed). *There is a large market for chelating agents for the treatment a variety of iron (and aluminium) overload conditions.* Novartis recently initiated a new programme for the development of orally-active iron chelators. They synthesised 744 compounds of which 3 have been selected for advanced trials based on their strong affinity for iron, oral activity and toleration. One is an hydroxypyridone, the second a tridentate ligand designed by computer, and the third a relative of HBED.

***General conclusions***
There are many basic areas of research into the Chemistry of Metals in Medicine that need to be explored and which have the potential to lead to major advances in health care. Such research is likely to be innovative and of high quality.
It should be recognised that traditional studies of organic drugs at a fundamental level are not complete without a parallel programme on metal pharmacology. Many organic drugs either require interactions with metals for activity, interact with metals at their target site or during their metabolism, or disturb the balance of metal ion uptake and distribution in cells and tissues. Indeed the use of organic agents (often chelating agents) to control the flux and distribution of metal ions is an exciting part of this new Action. Research programmes on organic and metallo-drugs should not be seen as mutually exclusive. They overlap extensively and the combination is likely to be a powerful force for the future.
It is apparent that metal compounds offer new properties that cannot be found amongst purely organic agents. In that respect they offer opportunities for the design of truly novel agents and for the treatment of previously intractable diseases and conditions.
It should be recognised that a metal is not just a metal: it is a metal ion plus its ligands. The metal ion and its ligands determine the biological activity.
In this field there are enormous opportunities for collaboration between academia and industry, and this can readily take place within the COST framework. Industries are ready to accept invitations to attend meetings of Working Groups when appropriate. The encouragement of close interactions between academia and industry is worthwhile.
The European scientific and technological research base for elemental medicinal chemistry is likely to be highly attractive for business.

---

*Enquiries about this report can be addressed to:*
*Professor Dr Peter J. Sadler*
*Chairman, COST D8*
*Department of Chemistry, University of Edinburgh*
*West Mains Road, Edinburgh EH9 3JJ, UK*

*Tel: +44-131 650 4729; Fax: +44-131-650 6452*
*E-mail: P.J.Sadler@ed.ac.uk*

